# Measuring adult Attention Deficit Hyperactivity Disorder using the Quantified Behavior Test Plus

**DOI:** 10.1002/pchj.17

**Published:** 2012-12-27

**Authors:** Hanna Edebol, Lars Helldin, Torsten Norlander

**Affiliations:** 1 Department of Psychology, Karlstad UniversityKarlstad, Sweden; 2 Department of Psychiatry, The NU-Health CareTrollhättan, Sweden; 3 Evidens Research and Development CenterGöteborg, Sweden; 4 Division of Psychology, Department of Clinical Neuroscience, Karolinska InstitutetSolna, Sweden

**Keywords:** adults, Attention Deficit Hyperactivity Disorder, psychometrics, objective measures

## Abstract

Attention Deficit Hyperactivity Disorder (ADHD) occurs in approximately 5% of the adult population and includes cardinal symptoms of hyperactivity, inattention, and impulsivity that may be difficult to identify with clinical routine methods. Continuous performance tests are objective measures of inattention and impulsivity that, combined with objective measures of motor activity, facilitate identification of ADHD among adults. The aim of the present study was to examine the sensitivity, specificity, and a composite measure of ADHD using objective measures of the ADHD-cardinal symptoms in adult participants with ADHD and non-ADHD normative participants. Cardinal symptoms were measured in 55 participants having ADHD, 202 non-ADHD normative participants, as well as 84 ADHD normative participants using the Quantified Behavior Test Plus. This test measures inattention and impulsivity using a continuous performance test, and hyperactivity using a motion-tracking system. A predictive variable for the detection of ADHD called Prediction of ADHD yielded 86% sensitivity and 83% specificity. A composite measure of ADHD cardinal symptoms was developed using a Weighed Core Symptoms scale that indicated the total amount of ADHD symptoms on a numeric scale from 0 to 100. The total amount of ADHD symptoms was measured on a scale and predicted with the categorical variable in a majority of the cases in the present study. Further studies are needed in order to confirm the results with regard to additional clinical and normative samples. Careful consideration of potential sex and diagnostic subtype differences are noteworthy aspects for future examinations of the new instruments.

In the 1950s, Continuous Performance Tests (CPT) were originally used for testing the signal detectability of radar operators. Development intended for psychiatric care and research eventually began in human subjects with brain damage (Rosvold, Mirsky, Sarason, Bransome, & Beck, 1956). From the early stages, neuropsychological performance deficits were the targets of much research because of the association with poor attention and impulse control. CPT measures were found to correspond with cardinal symptoms of Attention Deficit Hyperactivity Disorder (ADHD), that is, hyperactivity, inattention, and impulsivity (American Psychiatric Association [APA], 2000). The new measures, however, were merely considered for child and adolescent populations. Since then, the CPT paradigm has expanded both in terms of apparatus and techniques of testing. From merely being revolving drums on which letters are mounted side by side (Rosvold et al., 1956), it nowadays typically includes computerized presentation of visual or auditory stimuli at a rapid pace for a fixed time. The participant is instructed to respond to some stimuli, that is, targets, and to withhold response to other stimuli, that is, nontargets. What the CPT paradigm actually measures is based on clinical assumptions, expert judgments (Riccio & Reynolds, 2001), and face validity (Brocki, Tillman, & Bohlin, 2010). Omission errors are related to inattention and commission errors to impulsivity. Inattention may be defined as the incapacity to persist during a task, while impulsivity is the incapacity to inhibit an inappropriate response (Kaplan & Stevens, 2002).

Robust findings throughout the years are that children with ADHD typically identify fewer targets, respond to more nontargets and demonstrate decreased target detection with time (Charles, Schain, Zelniiker, & Guthrie, 1979; Corkum & Siegel, 1993; Douglas, 1972; Grodzinsky & Barkley, 1999; Losier, McGrath, & Klein, 1996; Pliszka, 1992; Riccio & Reynolds, 2001; Sykes, Douglas, & Morgenstern, 1973). Accumulated research demonstrates discriminative power for ADHD versus nonaffected controls, but the results are more inconsistent for ADHD versus other psychiatric disorders (McGee, Clark, & Symons, 2000; Riccio & Reynolds, 2001).

The paradigm has been developed with prime reference to children with ADHD, but is also administered frequently to adults with the disorder. As a result, a lack of sensitivity and ceiling effects have been documented when applying child norms to adult populations, particularly in cases with less severe symptomatology (Corbett & Stanzcak, 1999; Seidman, Biederman, Faraone, Weber, & Ouellette, 1997). When standardizing CPTs for adults, however, groups with ADHD have most often been identified as impaired compared with healthy controls. Recent meta-analytic reviews (Boonstra, Oosterlaan, Sergeant, & Buitelaar, 2005; Frazier, Demaree, & Youngstorm, 2004; Hervey, Epstein, & Curry, 2004) have reported medium to high effect sizes (Cohen's *d*) for many of the parameters of inattention and impulsivity. A meta-analytic review of neuropsychological measures (Schoechlin & Engel, 2005) found significant (*p* ≤ .01) pooled effect sizes for sustained attention (−0.52) on various CPTs, which placed them among the top discriminating measures of adult ADHD versus healthy controls.

Even though the CPTs have advanced in the past 60 years, they do not facilitate measures of hyperactivity. Instead, single- and multiple-channel actometry and motion-tracking systems have been used for recording motor activity in children and adults with the disorder. Children with ADHD demonstrate more frequent head-shifts, covering longer distances and greater areas, and with a more linear but less complex movement pattern in comparison to controls (Teicher, Ito, Glod, & Barber, 1996). Two studies (Boonstra et al., 2007; Tuisku et al., 2003) that used actometry in adults with ADHD or with ADHD and antisocial personality disorder reported higher frequencies of movements in ADHD as compared to nonaffected controls. The need for the activity domain to be objectively accessible and measurable in adult ADHD patients has been critically discussed, with reference to reduced levels of motor activity with increased age (Brocki et al., 2010) and the influence of diagnostic subtypes. However, the clinical definition of ADHD (APA, 2000) stipulates age-relevant symptoms of hyperactivity for diagnosis. Many adult persons with ADHD report fidgeting their feet and fingers (74%) and having problems remaining seated (66%) several times a day (Barkley, Murphy, & Kwasnik, 1996). Symptoms of inattention hold low specificity for ADHD and most studies with clinical controls report low discriminative power for conventional CPT measures. Hyperactivity is a distinct cardinal symptom of ADHD that is possible to measure in adulthood (Boonstra et al., 2007; Polcari, Fourligas, Navalta, & Teicher, 2010; Tuisku et al., 2003), and it may improve the ability to separate ADHD from psychiatric disorders with inattentive and impulsive symptom overlap.

Lis et al. (2010) were the first to investigate motor activity in adult persons with ADHD (*N* = 20) and compare them with healthy and matched controls (*N* = 20) using the Quantified Behavior Test Plus (QbTest-Plus). This test combines a motion-tracking system (MTS) and a CPT on the computer. It was developed for the purpose of reducing ceiling effects and to measure the ADHD core symptoms among adults. The highest separation of the groups was reported for the MTS measures that included parameters called microevents (the number of small motor shifts), time active (amount of time with motor activity), distance (*m*), and area (*m*^2^). With regard to the CPT measures, significant group differences were found for omission errors only. Motor activity was associated with increased levels of cognitive impairment in the ADHD group only. Motor activity in the ADHD group was up to 3.5-fold higher than that of healthy controls during the 20 min of standardized testing. The group differences became more accentuated as the period prolonged. Lis et al. (2010) suggested that, although observable motor behaviors might decrease with age, higher motor activity persists and may be recorded in adults with ADHD. Another study (Edebol, Helldin, & Norlander, in press) investigated the ability of the QbTest-Plus to discriminate ADHD from clinical controls with bipolar II disorder or borderline personality disorder and from participants who were assessed for but excluded from a diagnosis of ADHD. Objective motor activity measures were the most predictive marker of ADHD versus other clinical diagnoses, and the study provided support for composite measures of the disorder. Taken together, previous studies combining CPT and motor activity measures suggest an increased diagnostic accuracy and discriminative power for adult ADHD using combined and composite measures.

Despite access to simultaneous measurement techniques for the entire ADHD core symptom spectra, there is a lack of research regarding composite measures of the disorder. The present study wished to examine the possibility of combining measures of hyperactivity, inattention, and impulsivity from the QbTest-Plus into a composite measure for quantification of the disorder. The present study also wished to examine the predictive power of the QbTest-Plus in terms of its sensitivity and specificity for ADHD and non-ADHD normative participants. The aims of the present study were therefore twofold: first, to examine the predictive power of the QbTest-Plus in terms of its sensitivity and specificity for adult participants with ADHD and non-ADHD normative participants; and second, to develop a composite measure for the entire ADHD core symptom triad on the basis of the measures provided by the QbTest-Plus.

## Methods

### Participants

This study consisted of 341 participants, comprising three groups. The first were participants with ADHD (*N* = 55), the second were non-ADHD normative (*N* = 202), and the third were ADHD normative (*N* = 84).

#### The ADHD group

Demographic data for the ADHD group are presented in Table 1 and psychiatric data are presented in Table 2. Diagnostic assessments adhered to the *Diagnostic and Statistical Manual of Mental Disorders*, 4th edition (*DSM-IV*; APA, 2000). Clinical assessments had previously been carried out within two separate psychiatric clinics: the NU-Health Care in Västra Götaland, Sweden (*N* = 24) and the psychiatric division in the County Council of Värmland, Sweden (*N* = 19). Assessments included similar neuropsychological tests and procedures at both psychiatric clinics, such as tests of memory, attention, executive functioning, intelligence, interviews, observations, and somatic examinations. None of the participants used stimulants on the day of the QbTest-Plus, but 34 participants generally used stimulants. The approximate mean time from taking stimulant medication at the first minute of the QbTest-Plus was 30.55 hr (*SD* = 9.36 hr, range *=* 16–55 hr). A total of 22 persons did not use psychotropic medication, while 33 persons used one (*n* = 19), two (*n* = 10), three (*n* = 1), four (*n* = 2), or five (*n* = 1) psychotropic medications. Medications included antidepressants (*n* = 26), antiepileptics (*n* = 13), anxiolytics (*n* = 10), neuroleptics (*n* = 7), and anticholinergics (*n* = 1). The approximate mean time from taking psychotropic medication at the first minute of the QbTest-Plus was 16.27 hr (*SD* = 15.11 hr). Statistical analyses (independent samples *t*-test, 5% level) regarding age, education, Global Assessment of Functioning (GAF) total (Luborsky, 1962), GAF symptoms, GAF function, and age at first contact with psychiatry yielded no significant differences between the sexes (*p*s > .05). For means and standard deviations, see Tables 1 and 2.

**Table 1:** Demographic data for the ADHD group (*N* = 55)

Demographic	*n*	*%*	*M*	*SD*
Age	55		33.35	8.84
Gender				
Male	25	45.5		
Female	30	54.5		
Body Mass Index	55		26.18	4.94
Nicotine consumption				
No	23	41.8		
Yes	32	58.2		
Minutes from nicotine consumption when performing the Quantified Behavior Test Plus	32		38.19	27.43
Education				
Junior high school	12	21.8		
Partial high school	15	27.3		
Complete high school	19	34.6		
Partial graduate school	5	9.1		
Complete graduate school	4	7.3		
Employment status				
Sick leave	18	32.7		
Full- or part-time employment	13	23.6		
Rehabilitation/practice	7	12.7		
Unemployed	6	10.9		
Studying	5	9.1		
Retired	4	7.3		
Parental leave	2	3.6		
Income				
Public maintenance	38	68.7		
Employment	11	20.4		
Student loans	4	7.4		
Other income	2	3.7		
Social status				
Married/common-law	21	38.2		
Single	23	41.9		
Partner	11	20.0		
Household				
Single	24	43.6		
Shared	31	56.4		

*Note:* ADHD = Attention Deficit Hyperactivity Disorder.

**Table 2:** Psychiatric data for the ADHD group (*N* = 55)

Characteristic	*n*	*%*	*M*	*SD*
Age at first contact with psychiatry	55		21.46	9.75
Global assessment of functioning closest in time to the Quantified Behavior Test Plus	55		53.89	7.20
Function	55		54.59	8.68
Symptoms	55		54.49	6.86
Psychiatric hospitalization				
Never	24	43.6		
One time	20	36.4		
Several times	11	20.0		
Age at first hospitalization	31		24.86	7.80
Age at ADHD diagnosis	55		31.96	9.02
Diagnostic subtype				
ADHD-C	43	78.2		
ADHD-PI	12	21.8		
ADHD behavior rating scales				
ASRS total	55		44.58	10.98
ASRS screener	55		15.82	4.25
Wender Utah Rating Scale 25 most predictive	55		53.85	18.10
Wender Reimherr ADHD Symptoms Scale for Patients total	55		86.91	21.37
Conner's Adult ADHD Rating Scale total	55		47.25	12.14
Current psychiatric comorbidity				
None	24	43.6		
One	24	43.6		
Two	7	12.7		
Age at first comorbidity	31		31.96	8.99
Age at second comorbidity	7		29.86	5.81
Distribution of current comorbidity				
Substance abuse	7	18.4		
Relapsing/moderate depression	7	18.4		
Anxiety disorders	8	21.1		
Mixed anxiety/depression	2	5.3		
Bipolar disorders	6	15.8		
Personality disorders	4	10.5		
Adjustment disorders	2	5.3		
Autism	1	2.6		
Dyslexia	1	2.6		

*Note:* ADHD = Attention Deficit Hyperactivity Disorder; ASRS = ADHD Self-Report Scale.

#### The non-ADHD normative group

The second group of participants (*N* = 202) included 114 men and 88 women whose mean age was 31.06 years (*SD* = 10.27 years, range = 18–53 years). The group was recruited from the general population in order to represent non-ADHD normative participants and they did not have a diagnosis of ADHD. Participants were screened for symptoms of ADHD using the adult ADHD Self-Report Scale (ASRS; described in the instruments section). The mean total for the six items that were most predictive of ASRS, that is, the screener, was 8.90 (*SD* = 3.53), and the mean total overall was 24.56 (*SD* = 8.66). An independent samples *t*-test confirmed that the ASRS scores of the screener and the total were significantly different (*p*s < .001) from those of the ADHD group presented in Table 2. An independent samples *t*-test yielded no significant differences for men and women in the non-ADHD normative group with regard to age or ASRS (*p*s > .05).

#### The ADHD normative group

The third group of participants (*N* = 84) included 47 men and 37 women with ADHD whose mean age was 35.07 years (*SD* = 10.36 years). The participants had previously completed the QbTest-Plus as a part of a thorough neuropsychiatric assessment performed at the neuropsychiatric clinic, Cereb AB, in Stockholm, Sweden. Apart from the QbTest-Plus, the neuropsychiatric assessment also included tests of cognitive functioning, attention, intelligence, everyday functioning, and ADHD symptomatology.

The group was recruited for methodological purposes to represent an ADHD normative group during the development of the variable Prediction of ADHD (PADHD; see the instruments section) and the Weighed Core Symptoms scale (WCS; see the instruments section). For the PADHD, the ADHD normative data were employed to evaluate the levels of sensitivity and specificity. For the WCS, the ADHD normative data were employed in the assessment of core symptom manifestations and to examine the cut-off points on the scale. As this group was solely a normative reference group for ADHD in the present study, with the purpose of examining the PADHD and the WCS, findings from the group are not reported in the statistical analyses of the results section.

### Design

The present study had a quantitative design. Statistical analyses of variance and a Fisher's exact test were conducted using the software program Statistical Package for the Social Sciences version 19.0 for Windows (SPSS Inc., 2012). The independent variables of the present study were Group (non-ADHD normative group, ADHD-group) and Gender (men, women). There were 202 participants in the non-ADHD normative group and 55 participants in the ADHD group. The total number of men was 139 and the total number of women was 118. The dependent variables of the present study were the core ADHD symptoms of hyperactivity, inattention, and impulsivity, as well as the composite measure, the WCS scale. The categorical variable, PADHD, was analyzed using Fisher's exact test.

The dependent variables were developed statistically from the core symptom measures from the QbTest-Plus, that is, hyperactivity measured in distance, inattention measured using omission errors, and impulsivity measured using commission errors. The Q-score (Qb-test raw score) of each core symptom was transformed into percentages, where 0% indicated the maximum quantity of the symptom and 100% indicated the complete absence of the symptom. The additional scale, WCS, was constructed using weighed Q-scores from the symptom scales. The WCS runs from 0, which indicates the maximum amount of ADHD symptoms, and 100, which indicates a complete absence of ADHD symptoms. The weighed Q-score principle was based on analyses of Receiver Operator Curves, in which hyperactivity was the most predictive item of ADHD and therefore multiplied by 3, inattention was the second most predictive item and multiplied by 2, and impulsivity was ascribed no additional weight because of its relatively low predictive value.

The categorical variable, PADHD (no ADHD, yes ADHD), was also based on the raw scores from the measures of the QbTest-Plus (Q-scores), that is, hyperactivity measured in distance, inattention measured using omission errors, and impulsivity measured using commission errors. It was analyzed in the present study using Fisher's exact test (*p* < .05) with the PADHD (no ADHD, yes ADHD) and Group (non-ADHD normative group, ADHD group). For further information on how the PADHD was conducted see the instruments section.

### Instruments

#### QbTest-plus

This instrument (QbTech AB, 2010a, 2010b) combines a continuous performance test (CPT), installed as a software program on a PC, and an activity test performed over a 20-min period. While performing the CPT on the computer, the movements of the participants were recorded using an infrared camera following a reflective marker attached to a headband. The CPT test involves rapid presentations of four types of stimuli (a red circle, a blue circle, a red square, and a blue square) and the participants were instructed to press a handheld button when a stimulus subsequently repeated itself (a target) and not to press the button each time the stimulus varied relative to the previous one (a nontarget). The stimuli were presented at a rate of one per 2 s, with each one visible for 200 ms, and the total number of stimuli was 600, presented with a 25% target probability. The purpose of the QbTest-Plus is to provide objective information regarding cardinal symptoms of ADHD: hyperactivity assessed on the basis of motor activity measured using the camera, and inattention and impulsivity measured on the basis of the CPT (QbTech AB, 2010b).

In this study, hyperactivity has been operationalized with the parameter called “distance,” that is, the length of the path describing the movement of the headband reflector during the test. Inattention has been operationalized on the basis of omission errors, that is, when no response is registered and the stimulus was a target. Finally, impulsivity has been operationalized on the basis of commission errors, that is, when a response was registered and the stimulus was a nontarget. A former study of the QbTest-Plus (Lis et al., 2010) demonstrated the relative significance of the core symptoms, with measures of hyperactivity being the most and inattention the second-most efficient measure of ADHD, whereas the level of impulsivity was not significantly higher in adults with ADHD compared with healthy controls (*p* > .05).

In this study, the relative significance of the core symptoms was taken into consideration by the means of the WCS, which weighs the scales of the core symptoms into one measure. The WCS scale was derived from the ADHD group and the non-ADHD normative group of the present study, and was tested on the normative group with ADHD, as described in the participants section. The ADHD normative group was employed in the assessment of core symptom manifestations and to examine the cut-off points on the scale. The best model adjustment was found when the results of the core symptoms were summed after the results of hyperactivity had been multiplied by 3, the results of inattention had been multiplied by 2, and the results of impulsivity by 1. Then, the total results from the ADHD group of the present study, the ADHD normative group, as well as the non-ADHD normative group, were categorized using 10 cut-off points (visual binning, *With* = 9.09%., i.e., each individual could gain one out of eleven sums on the scale). In the present study, the WCS correlated (Pearson's *r*) with hyperactivity (*r* = .76, *p* < .001), inattention (*r* = .72, *p* < .001), and impulsivity (*r* = .54, *p* < .001), and those results were also approximately the same when correlations were computed only for the ADHD group or only for the non-ADHD normative group.

The categorical variable, PADHD (no ADHD, yes ADHD), was based on qualitative analyses of raw scores from the operationalized measures of the QbTest-Plus (Q-scores). Data were derived from the ADHD group and the non-ADHD normative group of the present study. The qualitative analyses included testing the effects of setting various cut-off points as well as combining and coordinating cut-off points from the core symptom measures of hyperactivity (distance), inattention (omission error), and impulsivity (commission errors). The effects of the cut-off points were evaluated in terms of sensitivity (positive predictive value) and specificity (negative predictive value) for the ADHD group and the non-ADHD normative group. Multiple cut-off points for each of the three core symptoms combined with the coordinated cut-off points from the other two core symptoms resulted in multiple pathways to either of the two categories of PADHD (no ADHD, yes ADHD). Except for distance, omission errors, and commission errors, the other parameters from the QbTest-Plus were assessed in terms of sensitivity and specificity, but according to the aggregated assessment trials, the most efficient parameters for the identification of ADHD and separation from non-ADHD normative participants were the three parameters that were ultimately included in the PADHD. Apart from the coordinated cut-off points for distance, omission errors, and commission errors, the PADHD also included supplementary cut-off points from a parameter called the normalized variation Q-score (NVQ). This variable is a mathematical measure defined as the standard deviation of the reaction time divided by the reaction time. It was included in the PADHD because it improved the predictive power. The NVQ is not a principal parameter in the PADHD, but is a supplementary parameter for the three core symptom measures.

In order to make sure that the PADHD was not simply an adjustment for the ADHD group and the non-ADHD normative group of the present study, the PADHD was evaluated in terms of sensitivity and specificity for the ADHD normative group described in the participants section of the present study. The level of sensitivity became somewhat higher for the ADHD normative group than the ADHD group of the present study. This gave credit to the PADHD as not being an adjustment to the ADHD group of the present study. After the PADHD had been conducted through qualitative analyses and evaluated on the ADHD normative group described in the participants section, it was analyzed statistically for the present study using Fisher's exact test (*p* < .05) for Prediction of ADHD (no ADHD, yes ADHD) and Group (non-ADHD normative group, ADHD group).

#### Global Assessment of Functioning Scale

The Global Assessment of Functioning (GAF) Scale (Luborsky, 1962) can be found in the *DSM-IV* (APA, 2000), and estimates psychological, social, and occupational functioning on a numeric continuum (0–100) of mental health and illness for adult persons. The first interval (0–50) describes severe symptoms, whereas the second interval (50–100) describes functional disabilities. The total score (GAF-total) of this scale should be the lowest score of either the symptoms (GAF-symptom) or the functional disability score (GAF-function). The GAF-scores presented in the current study were calculated by licensed clinicians and were the scores reported in the psychiatric records closest in time to the QbTest-Plus.

#### The Adult Self Report Scale for adult ADHD v1.1

The Adult Self-Report Scale (ASRS) is a screening instrument (Kessler et al., 2005; Kessler & Üstün, 2005) that was developed by the World Health Organization in order to provide initial information about the prevalence of ADHD symptoms for both research and health-care centers. It is derived from the criteria for ADHD in the *DSM-IV*. Part A includes the six most predictive items while part B holds an additional 12 items, all rated on a 5-point scale (0 = never, 1 = rarely, 2 = sometimes, 3 = often, and 4 = very often). Each item has a cut-off point of either 2 (sometimes) or 3 (often), and a result of four or more items listed above the cut-off point in part A is used as an inclusion/exclusion criterion for clinical purposes and in research programs, such as in the normative sample of the Qbtest-Plus (QbTech AB, 2010a). The internal consistency for the patient-administered version is.88 (Cronbach's alpha; Kessler et al., 2005).

### Procedure

**Figure 1:**
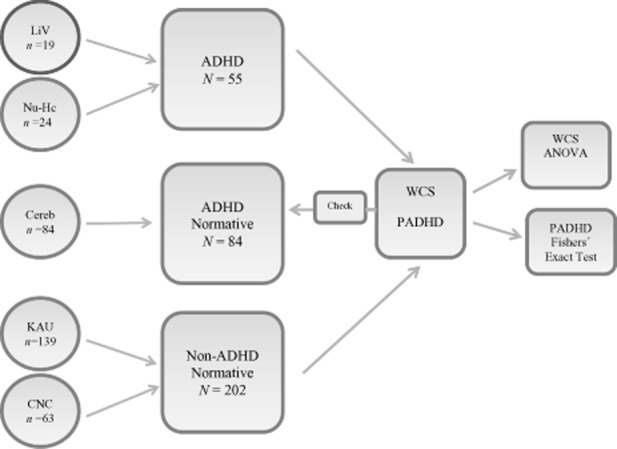
A presentation of the recruitment and analyses of data in the present study. ADHD = Attention Deficit Hyperactivity Disorder; PADHD = Prediction of ADHD; WCS = Weighed Core Symptoms scale.

Since 2008, the Department of Psychology at Karlstad University has been participating in a research program regarding the objective markers of adult ADHD. The research program includes studies on both the diagnostic and treatment aspects of the disorder. It is organized as collaboration between the Department of Psychology at Karlstad University, Sweden, the Cognitive Neuroscience Centre for Psychiatry at the University of Giessen, Germany, the psychiatric clinic in the NU-Health Care in Västra Götaland, Sweden, the psychiatric division of the County Council of Värmland, Sweden, and the neuropsychiatric clinic Cereb AB in Stockholm, Sweden. A presentation of the process for recruitment and analysis is given in Figure 1. The study procedures were examined and approved by the Regional Ethical Review Board of Uppsala, Sweden, in February 2008.

#### The ADHD group

Nurses and trained clinicians at the psychiatric centers screened psychiatric records and recruited participants via mail and telephone. The vital inclusion criteria for the ADHD group were an age of 18–65 years, a diagnosis of ADHD according to the *DSM-IV* (APA, 2000), a described chronic course of ADHD symptomatology from childhood to adulthood with some symptoms present before 7 years of age and continuing to meet the *DSM-IV* criteria at the time of assessment, and an accepted withdrawal from central stimulant treatment 24 hr prior to the QbTest-Plus. Exclusion criteria for this group were any clinically unstable psychiatric condition including, but not limited to, acute mood disorder, acute bipolar disorder, and acute obsessive-compulsive disorder (OCD), and not meeting the diagnostic criteria for ADHD in the *DSM-IV*. The study-specific procedures were explained and information about withholding one's diagnostic and medical status from the researcher as well as withdrawing from central stimulant treatment 24 hr prior to participation were highlighted in particular. On the day before participation, the participants received a short message on their mobile phone that included a reminder of the day, time, and location of the study.

When arriving at the psychiatric center, the participant was met by a researcher in the lobby and taken to the test room, where information about the study procedures was provided. The written consent form was then introduced, and a signed copy received from the participant. The ASRS and a medical form, regarding the paticipant's medical status and the last time that central stimulant treatment was used, were then filled out. The patient's diagnosis and status was unknown to the researcher during the whole process of experimental testing and assessment. Prior to testing, the participant was seated on a chair without armrests in order to ensure a nonreclining body position and also provided with the reflective head-band and the handheld button.

Instructions on how to do the QbTest-Plus were given first verbally and then by the means of a standardized video (QbTech AB, 2010b) that presented the procedures for the QbTest-Plus. The participant performed a 1-min pretest to make sure the instructions had been understood correctly. After this, the participant performed the QbTest-plus for 20 min. A room with minimal visual and auditory stimuli was used for testing. After testing, the participant answered demographic and other relevant questions regarding his or her psychiatric history, but no questions that endangered the blind design were asked. For the purposes of analysis, data covering the results and methods used for clinical assessment, psychiatric history, the latest GAF, and information about potential medical treatment at the time of the study were collected from the participants' clinical records. Four participants with ADHD were excluded from the analysis because of central stimulant treatment during participation (*n* = 3) and because of not obtaining the diagnostic requirements regarding ADHD (*n* = 1), which resulted in a total number of 55 participants for this group.

#### The non-ADHD normative group

A majority of the participants in the non-ADHD normative group (*n* = 139) were recruited with the assistance of students at the Department of Psychology at Karlstad University in Karlstad, Sweden. The remaining part of the group (*n* = 63) was recruited with the assistance of the Cognitive Neuroscience Centre for Psychiatry at the University of Giessen, Germany. In Karlstad, recruiting was performed using major employers in the fields of education (*n* = 48), transportation/industry (*n* = 44), as well as among students at the University (*n* = 47). In Giessen, all recruiting was performed via major music organizations. The inclusion criteria for the normative group were an age of 18–65 years and a willingness to sign the informed consent and complete the study-specific procedures. The exclusion criterion was any known psychiatric diagnosis. The QbTest-Plus was performed at the Universities of Karlstad and Giessen. The procedures for experimental testing with the QbTest-Plus were the same as described above for the ADHD group.

#### The ADHD normative group

The participants in this group had previously completed the QbTest-Plus as part of a thorough neuropsychiatric assessment at the neuropsychiatric clinic Cereb AB in Stockholm, Sweden. The clinical assessments adhered to the diagnostic criteria outlined in the *DSM-IV* (APA, 2000). The unstandardized test report from the QbTest-Plus served as a basis for the diagnostic evaluation, along with other instruments of clinical assessment. Apart from the QbTest-Plus, neuropsychiatric assessments included for example tests of cognitive functioning, attention, intelligence, ADHD symptomatology, and everyday functioning. The group was recruited with the assistance of Cereb AB for methodological purposes, in order to represent an ADHD normative group during the examination of the instruments developed in the present study, that is, the PADHD and the WCS. Because this group is solely a normative reference group for ADHD in the present study, it is not reported in the statistical analyses of the results section of the present study. The procedures for experimental testing with the QbTest-Plus were the same as described above for the ADHD group.

## Results

### Prediction of ADHD

Fisher's exact test (5% level) with group (non-ADHD normative group, ADHD group) and the categorical variable, PADHD (No, Yes), showed a strong significant connection (*p* < .001) between the variables, indicating a vast majority for No in the non-ADHD normative group and a vast majority for Yes in the ADHD group, as well as a Yes minority in the non-ADHD normative group and a No minority in the ADHD-group. The effects of the sensitivity and specificity for the categorical variable of the present study are presented in Table 3.

**Table 3:** Effects of Prediction of ADHD (No ADHD, Yes ADHD) for the Non-ADHD Normative (*N =* 202), and ADHD (*N =* 55) Groups

	Prediction of ADHD			
	
	No		Yes	
		
Group	Frequency	%	Frequency	%
Non-ADHD normative	167	83	35	17
ADHD	8	14	47	86

*Note*: There was a significant connection between prediction of ADHD and group. ADHD = Attention Deficit Hyperactivity Disorder.

### Group and gender differences with regard to dependent variables

A Pillai's MANOVA (2 × 2 factorial design) was conducted with group (non-ADHD normative group, ADHD group) and gender (men, women) as independent variables, and hyperactivity, inattention, and impulsivity as dependent variables. The analysis revealed significant effects for group (*p* < .001, η^2^ = .31, power >.99) and for gender (*p* = .005, η^2^ = .05, power = .86). However, the analysis did not show a significant main effect for the interaction of group and gender (*p* = .54, η^2^ = .008, power = .20). The results from the univariate *F*-tests regarding group and gender are given below.

#### Core symptoms

Univariate *F*-tests revealed significant effects of group for hyperactivity, *F*(1, 253) = 31.53, *p* < .001, Inattention, *F*(1, 253) = 70.00, *p* < .001, and Impulsivity, *F*(1, 253) = 47.45, *p* < .001, where the non-ADHD normative group scored higher on all variables compared with the ADHD group. With regard to gender, univariate *F*-tests revealed a significant effect for inattention, *F*(1, 253) = 8.05, *p* = .005, but not for hyperactivity or impulsivity (*p*s > .05). Further analysis showed that women scored lower on inattention, indicating higher symptom levels, compared with men. For means and standard deviations see Table 4.

**Table 4:** Means and Standard Deviations for Hyperactivity, Inattention, Impulsivity, and the WCS with Regard to Group (Non-ADHD Normative Group, *N =* 202; ADHD Group, *N =* 55) and Gender

	Non-ADHD normative group				ADHD group			
		
	Men (*n* = 114)		Women (*n* = 88)		Men (*n* = 25)		Women (*n* = 30)	
				
	*M*	*SD*	*M*	*SD*	*M*	*SD*	*M*	*SD*
Hyperactivity	73.41	13.23	71.85	13.81	60.72	22.48	56.37	26.04
Inattention	57.63	15.99	52.67	14.17	40.20	15.22	32.37	10.42
Impulsivity	73.10	14.35	74.18	15.45	50.44	31.31	57.57	26.44
WCS	66.40	25.10	61.25	26.34	33.20	26.57	26.67	21.23

*Note*: The non-ADHD normative group differed from the ADHD-group in all respects. There was no difference between men and women except for inattention. There was no interaction between group and gender. ADHD = Attention Deficit Hyperactivity Disorder; WCS = Weighed Core Symptoms scale.

#### Weighed Core Symptoms scale

As the WCS is a composite measure, it needed to be analyzed separately using a two-way ANOVA. The analyses showed a significant effect of group, *F*(1, 253) = 77.10, *p* < .001, but not of gender or the interaction of group and gender (*p*s > .05). The descriptive analyses showed that the non-ADHD normative group scored higher on the WCS compared with the ADHD group. For means and standard deviations see Table 4.

### Differences of prediction of ADHD with regard to dependent variables

Subsequent analyses were conducted using a one-way Pillai's MANOVA with the categorical variable, PADHD (no ADHD, yes ADHD), to investigate the relations with hyperactivity, inattention, and impulsivity. The analysis revealed significant results with PADHD, *p* < .001, η^2^ = .51, power > .99. The results from the univariate *F*-tests are given below.

#### Core symptoms

Univariate *F*-tests revealed significant effects of hyperactivity, *F*(1, 255) = 72.30, *p* < .001, inattention, *F*(1, 255) = 144.93, *p* < .001, and Impulsivity, *F*(1, 255) = 64.37, *p* < .001, where the no ADHD group scored higher on all variables compared with the yes ADHD group. For means and standard deviations see Table 5.

**Table 5:** Means and Standard Deviations for Hyperactivity, Inattention, Impulsivity, and the WCS with Regard to Prediction of ADHD (No ADHD, Yes ADHD)

	No ADHD (*n* = 175)		Yes ADHD (*n* = 82)	
		
	*M*	*SD*	*M*	*SD*
Hyperactivity	75.24	11.52	57.73	21.45
Inattention	58.26	14.64	36.40	10.92
Impulsivity	75.63	12.73	56.21	26.12
WCS	70.69	21.48	27.07	18.69

*Note*: The No group differed from the Yes group in all respects. ADHD = Attention Deficit Hyperactivity Disorder; WCS = Weighed Core Symptoms scale.

#### Weighed Core Symptoms scale

The WCS was again analyzed separately using a one-way ANOVA. The analyses showed a significant effect for PADHD, *F*(1, 255) = 249.34, *p* < .001, and descriptive analyses also showed that the group which was not supposed to have ADHD, according to the prediction variable, scored higher on the WCS compared with the group that was predicted to have ADHD. For means and standard deviations see Table 5.

## Discussion

The aims of the present study were twofold. First, to examine the predictive power of the QbTest-Plus in terms of its sensitivity and specificity for ADHD in adult persons, and second, to develop a composite measure for the entire ADHD core symptom triad on the basis of the measures provided by the QbTest-Plus. Two psychometric measures were developed in the present study. The first measure was the WCS, which quantified the amount of ADHD cardinal symptoms. The second measure was PADHD, which correctly classified ADHD in a majority of the cases. In this section, the results of the present study will be discussed in relation to some of the existing literature. First, the results from the examination of the sensitivity and specificity will be discussed, followed by the composite measure, and finally the limitations of the present study will be considered.

### Sensitivity and specificity

In line with another recent study of PADHD (Edebol et al., in press), most of the ADHD participants and the non-ADHD normative participants in the present study were correctly classified using the PADHD, as reflected in 86% sensitivity and 83% specificity. Apart from ADHD, the previous study of the PADHD (Edebol et al., in press) also included groups of participants with bipolar II disorder or borderline personality disorder and participants assessed for but excluded from a diagnosis of ADHD, as well as normative participants without ADHD. In that study, the PADHD yielded 87% sensitivity and 85% specificity for non-ADHD normative participants, which strengthens the findings of the present study. Another study (Edebol, Helldin, Holmberg, Gustafsson, & Norlander, 2011) investigated the predictive power of the QbTest-Plus using standardized cut-off points (Q > 1.3) among participants that were being assessed for ADHD. The analyses with the cut-off points yielded 83% sensitivity and 57% specificity. However, some of the participants in that study (Edebol et al., 2011), as well as in the present study, had psychiatric disorders in addition to ADHD, which makes interpretation of the results somewhat limited. In this context, the 87% sensitivity found in the ADHD group with minimal psychiatric comorbidity (Edebol et al., in press) both replicates the findings of the present study and supports the validity of the results as being associated with ADHD, rather than a psychiatric symptom load in general. The results for the PADHD in the present study are thus confirmed by the findings on well-defined ADHD samples without severe comorbidity (Edebol et al., in press). The fact that other clinical groups with bipolar II, borderline, and disconfirmed ADHD yielded lower levels of sensitivity (i.e., 36–41%) than the ADHD group (Edebol et al., in press) also strengthens the result from the present study of the PADHD being associated with ADHD more than with psychiatric disorders sharing symptoms with ADHD. A contribution of the present study when it comes to estimating the predictive power of the QbTest-Plus is that PADHD was evaluated on a well-assessed and convincing pool of patients with ADHD. The clinical assessments were thorough, well-documented, and completed prior to experimental testing. Most of the cases were correctly classified using the PADHD despite the additional psychiatric disorders. As ADHD is commonly associated with additional psychiatric disorders that may confound clinical assessment, the predictive power in terms of both sensitivity and specificity are vital for psychometric instruments investigated for clinical practice.

The results of the QbTest-Plus in the present study may be compared with similar instruments in terms of sensitivity and specificity. Walker, Shores, Trollor, Lee, and Sachdev (2000) investigated the predictive values of frequently used neuropsychological tests for adults with ADHD. Significant effects were found for the number of omission errors on the CPT and a few other neuropsychological tests. The predictive values for omission errors with regard to healthy controls were 63% sensitivity and 90% specificity, and with regard to clinical controls they were 63% sensitivity and 60% specificity. The level of sensitivity found for ADHD in the Walker et al. study (i.e., 63%) was relatively low compared with the present study, but the level of specificity for the nonaffected participants (i.e., 90%) was in line with the present study. Walker et al. found the most prominent aspect of ADHD during CPT testing to be inattention, as measured using omission errors. In the general literature, attention impairments have consistently been reported during the CPT performance of adults with ADHD (Johnson et al., 2001), whereas impulsivity has been less consistent (Losier et al., 1996). Likewise, in the present study, inattention was the most common and impulsivity the least common feature of ADHD performance. The most specific feature of ADHD in our study, regardless of the diagnostic subtype, was motor activity, which is also consistent with studies that have applied MTS or actigraphy measures in order to operationalize hyperactivity as motor activity (Boonstra et al., 2007; Lis et al., 2010; Polcari et al., 2010; Teicher et al., 1996).

Another large-scale study (Woods, Lovejoy, Stutts, Ball, & Fals-Stewart, 2002) combined several neuropsychological measures of verbal fluency, cognitive flexibility, divided attention, information processing speed, freedom from distractibility, and the recall component of word list learning in order to increase the diagnostic accuracy for adult ADHD. Depending on the cut-off point, sensitivity ranged from 73–85% and specificity from 69–77%. However, these predictive values are generally somewhat lower or similar to that of the PADHD in the present study. Recently, a systematic review was conducted for the psychometric properties of the most common self-rating scales for adult ADHD (Taylor, Deb, & Unwin, 2011). The review found Conner's Adult ADHD Rating Scale (CAARS) to be one of the most accurate scales, presenting 82% sensitivity and 87% specificity. Another common scale, the Adult ADHD Self-Report Scale (ASRS) had among the better psychometric properties of all the investigated scales, presenting 39–69% sensitivity and 88–100% specificity. Compared with the literature on instruments and screening methods for adult ADHD, the predictive values of the PADHD in the present study suggest that a laboratory measure applying combined CPT and MTS presents similar or better psychometric properties than commonly used screening instruments for adult ADHD. Moreover, recent studies (Walker et al., 2000; Woods et al., 2002) also suggest that the predictive values reported for the PADHD in the present study are more reliable for the correct classification of ADHD than solitary CPT measures. Finally, a combination of CPT and MTS measures like the one investigated in the present study provides unique aspects of cognitive as well as psychomotor aspects of the disorder, which is both psychometrically useful and characteristic of the disorder in adulthood (Boonstra et al., 2007; Lis et al., 2010; Polcari et al., 2010; Teicher et al., 1996). Further diagnostic and treatment implications may therefore be investigated.

### The composite measure

The second aim of the present study was to develop a composite measure for the entire ADHD core symptom triad on the basis of the measures provided by the QbTest-Plus. This was possible by means of the WCS. The scale separated the majority of the participants with ADHD from the majority of the participants without ADHD. The participants with ADHD had lower scores on the WCS (indicating higher levels of total symptoms) than the participants without ADHD, who had higher scores on the WCS (indicating lower levels of total symptoms). The WCS was developed using Qb-Test raw scores from the cardinal symptom measures of hyperactivity, inattention, and impulsivity. The scale was transformed, using visual binning with ten cut-points, into percentages where 0 indicated the maximum amount of ADHD cardinal symptoms and 100 indicated the complete absence of the symptoms. Interestingly, hyperactivity was the measure with the most diagnostic specificity, occurring frequently and most exclusively in the ADHD group regardless of the diagnostic subtype. Inattention was the most common symptom for both groups, which gives this measure a particular significance for diagnostic sensitivity. Impulsivity is probably the measure with the least sensitivity. It occurred infrequently in the ADHD group, but when it did so, it was often in extreme forms and usually along with other cardinal symptoms, consequently not adding any exclusive information for the detection of ADHD, altogether rendering low specificity. This relative significance of the cardinal symptoms was emphasized when constructing the WCS, because symptoms were given various weights, which is in line with the literature on the implications of core symptoms for the clinical diagnosis of ADHD (Murphy & Barkley, 1996).

In line with previous research (Edebol et al., in press; Lis et al., 2010; Polcari et al., 2010), both the raw scores and the psychometric instruments of the present study confirmed that the level of subtle motor activity was highly marked in subjects with ADHD compared with subjects without the disorder. Inattention was another strong marker of ADHD, but not enough to identify and separate a significant proportion of the ADHD group from the non-ADHD normative group. Conventional CPT measures of inattention and impulsivity have previously been reported to lack high enough levels of sensitivity and specificity to correctly classify ADHD among adults (Corbett & Stanzcak, 1999; Seidman et al., 1997; Walker et al., 2000; Wood et al., 2002). Importantly, neither of the core symptom measures in the present study solely identified a significant number of subjects with ADHD. Only the composite measure generated significant differences because, unlike the core symptom measures, the WCS is calibrated with the entire clinical ADHD phenomenon and the relative impact of behavior manifestations.

The present study did not intend to investigate the PADHD and the WCS across the diagnostic subtypes of ADHD. Thus, the sample was not selected to be representative of the clinical phenomenon with regard to its relative proportions of diagnostic subtypes. However, it is worth mentioning that the majority of the ADHD participants in the present study were correctly classified using the PADHD and marked as impaired on the WCS regardless of their diagnostic subtype. The proportion of the ADHD predominantly inattentive subtypes in the present study (22%) was, however, lower than the clinical prevalence in general, that is, 37% (Millstein, Wilens, Biederman, & Spencer, 1997). The low total number of participants with ADHD in general (*n* = 55) and with the inattentive subtype in particular (*n* = 11), suggests that additional studies with more representative samples are needed in order to investigate the present results with regard to the diagnostic subtypes of the clinical population with ADHD in general. As the hyperactivity measure has been emphasized in the present study, it is vital to investigate the levels of sensitivity with regard to well-defined and clinically representative samples of the ADHD diagnostic subtypes.

Even though the PADHD and the WCS are dependent on the same QbTest-raw scores, they were developed independently of each other. Statistical analyses with the PADHD and the WCS indicate that negative prediction was related to higher scores on the scales (indicating lower symptom levels), whereas positive prediction was related to lower scores on the scales (indicating higher symptom levels). Consequently, measures of ADHD (the WCS) and prediction of ADHD (the PADHD) were associated with each other and with actual ADHD (clinical diagnosis) in a majority of cases.

The present study also developed three separate core symptom scales intended for the patient and the clinician to use when illustrating and discussing symptom severity, as well as for monitoring treatment programs and progression. The core symptoms scales and the composite scale represent dimensional approaches to ADHD diagnosis and treatment. The behavioral measures are in line with the current clinical perspectives on ADHD and enable quantification of symptom severity and validation of subjective behavior ratings made by patients and family members. Both from the empirical scientific quarter and from literature reviews of adult ADHD (Epstein, Johnson, Varia, & Conners, 2001) using recent neuropsychological and neuroimaging perspectives, a heavier weight on performance-based assessments has been suggested for clinical and research methodologies (Doyle et al., 2005; Frazier et al., 2004). This would preferably include methodologies with empirically derived and age-sensitive neuropsychological tests and behavioral measures that potentially differentiate ADHD from other psychiatric disorders with shared symptoms with regard to ADHD.

The results of the present study suggest that the behaviors of ADHD may be communicated with the WCS, which is a composite measure of ADHD. The WCS was constructed in order to recognize the levels of malfunctioning related to ADHD and, as such, it may be used for diagnostic purposes. But a continual measure of ADHD communicates not only degrees of dysfunction, but also, it is hoped, positive improvements made during treatment programs and clinical interventions. And to better understand the true meaning of a certain measure, experiences of personal development and management are essential parts of the interpretation.

Cognitive and behavioral aspects of ADHD seem to occur on a continuum, and diagnostic recognition is being defined in clinical rather than statistical terms. According to the WCS it seems meaningful to look at the continuum across groups participating in the current study, not only for the purpose of finding an optimal cut-off point, but to gain a perspective of the broader formations of the disorder as well. The prevalence of ADHD in the adult normal population has previously been reported to be approximately 5% (DuPaul et al., 2001; Murphy & Barkley, 1996). This may, however, be restricted due to the diagnostic requirements being fixed across the lifespan. Assuming instead the principle that ADHD exists on a continuum, the prevalence of ADHD in the non-ADHD normative group of the present study may indicate sensitivity also towards the intermediate zones on the continuum. A full 100% specificity is perhaps not attainable, even when using an “objective” measure of ADHD. Perhaps the continuum of the current study illustrates that the term “objective” relates to the standardized procedures of the test and the unbiased interpretations of the measures rather than a 100% precise test of the disorder. The non-ADHD normative group was less well investigated than the ADHD group, however, and accordingly its characteristics, such as the educational, social, and employment status, were not fully identified, so the level of match with the ADHD group is unclear. However, as opposed to reporting the standardized cut-off points for the test or taking into account only the isolated symptom measures, the present study suggests that more accurate interpretations of ADHD are facilitated when standardized interpretations like the PADHD and the composite WCS are applied, which is also in line with the test objectives.

In order to develop standardized interpretations of the WCS more suitably, the gender aspect could be taken into consideration, as the present study as well as another study (Edebol et al., in press) observed that women generally showed statistically significant higher levels of inattention than men. This finding may be explained, for example, by a bias in the matching or selection criteria for the groups, or it may be an artifact of diagnostic tendencies for women being more dysfunctional than for men when receiving a diagnosis of ADHD, which is also reflected in the current male-referenced diagnostic standards (Barkley, 2006). The finding is, however, not consistent with other studies of CPT performance in ADHD (Gaub & Carlson, 1997; Gershon, 2002; Newcorn et al., 2001), where instead women are found to exhibit less or similar degrees of inattention then men. Prospective studies of the QbTest-Plus should be attentive to potential gender effects and biases so that the standards should be adjusted accordingly if the observations are confirmed. Also, the WCS and the PADHD should be developed with regard to treatment and clinical intervention programs so that additional tests and measures of positive improvement may be taken into account for future studies.

### Limitations

The limitations of the present study concern the comorbid psychiatric disorders in the group with ADHD. Most psychiatric disorders are potential biases when measuring attention, impulsivity, and even motor activity. The proportion of participants with the ADHD predominantly inattentive subtype was 22%, which is slightly low for clinical samples of adult ADHD in general. Another obvious limitation concerns the potential bias of psychotropic medication. Yet another limitation concerns the non-ADHD normative group because participation was based on personal interest, which may contribute to a sampling bias. Finally, it is not clear to what degree the non-ADHD normative group is characteristic of the population in general, and the lack of certain demographic data for this group also limits the comparison with the ADHD group.

## Summary

The Quantified Behavior Test Plus was examined with regard to ADHD cardinal symptoms. Hyperactivity, measured in motor activity, was the most specific symptom of ADHD. Inattention was the most sensitive and impulsivity the least sensitive symptom of ADHD when measured with a continuous performance test. ADHD was measured on a 10-point composite scale called the Weighed Core Symptoms scale, and predicted using the variable Prediction of ADHD in the majority of cases. The participants with ADHD had lower scores on the WCS (indicating higher levels of total symptoms), and participants without ADHD had higher scores on the WCS (indicating lower levels of total symptoms). The predictive values were similar to or higher than traditional measures of ADHD, such as behavior rating scales and neuropsychological tests. The study had limitations concerning psychiatric comorbidity and psychotropic medication in the group with ADHD, and some demographic data was not available to facilitate the matching process of the non-ADHD normative group.
